# Enhancing Airtight Storage with Germinating Cowpea Seeds: Impacts on Insect Mortality, Progeny and Grain Quality

**DOI:** 10.3390/insects14120954

**Published:** 2023-12-15

**Authors:** Gunakeshari Lamsal, Dieudonne Baributsa

**Affiliations:** Department of Entomology, Purdue University, 901 W. State St., West Lafayette, IN 47907, USA; glamsal@purdue.edu

**Keywords:** cowpea weevil, stored grains, hermetic storage, moisture content

## Abstract

**Simple Summary:**

Hermetic (airtight) storage systems are used by smallholder farmers to mitigate storage losses caused by insects. These systems rely on insects to consume the residual oxygen within containers. However, prolonged exposure of infested grains to oxygen during hermetic storage can further exacerbate damage. While commercial oxygen scavengers can eliminate residual oxygen, they are costly, unavailable, and unsuitable for smallholder farmers in developing countries. We investigated the potential of germinating cowpea seeds (10, 20, and 30 seeds) as oxygen scavengers within 2 L airtight storage jars filled with cowpea grains. A set of jars was opened daily for 5 days to evaluate insect mortality, progeny, and grain quality. We observed a 100% insect mortality in 72 h when cowpea weevils were exposed to low oxygen levels from 20 and 30 germinating seeds. Infested grains exposed for 96 and 120 h to hypoxia from 30 and 20 germinating seeds, respectively, exhibited no post-treatment progeny development. For optimal results, a 2 L hermetic container filled with infested cowpeas containing 20 or 30 germinating cowpea seeds used as oxygen scavengers should be closed for 5 days.

**Abstract:**

Millions of smallholder farmers use airtight (hermetic) storage to preserve stored commodities. However, relying on biological agents (i.e., insects) to deplete residual oxygen in airtight containers can occasionally extend the process, potentially resulting in grain damage or nutrient loss. Current oxygen scavengers used to remove this residual oxygen are unavailable and unsuitable on smallholder farms in developing countries. We evaluated the effectiveness of germinating seeds for oxygen depletion. Treatments comprised 10, 20, and 30 germinating cowpea seeds in 2 L jars filled with infested cowpea grains. Insect mortality and grain quality were assessed after 24, 48, 72, 96, and 120 h. Progeny development was monitored for 49 days post-treatment. The results showed that all germinating seeds depleted oxygen to 5% or below within 48 h. Complete adult mortality was achieved after 72 h for both 20 and 30 germinating seeds and 120 h for 10 germinating seeds. As germinating seeds increased, egg counts decreased. No adults emerged post-treatment after insects were exposed for 96 and 120 h to hypoxia from 30 and 20 germinating seeds, respectively. However, 120 h insect exposure to hypoxia from 10 germinating seeds had negligible progeny development. Moisture content increased slightly in grains exposed to 30 germinating seeds. Germinating seeds are as effective as controlled atmospheres in accelerating insect deaths, but further research is needed for field application and their effects on stored product quality.

## 1. Introduction

*Callosobruchus maculatus* (Coleoptera: Bruchidae), commonly known as cowpea weevil, is a devastating pest of legumes, particularly in subtropical countries. These beetle species cause substantial damage to stored grains such as cowpeas, chickpeas, peas, green gram, lentils, common beans, and other legume crops [1]. This species’ larvae are more destructive than adults, as the adults do not feed [2]. Female weevils lay their eggs on the surface of seeds, and upon hatching, the larvae tunnel into the seeds, leading to a significant loss in seed weight, germination viability, and market value of stored products [3,4,5].

Smallholder farmers use various storage methods to protect cowpea grains from insect damage, including granaries, ash, sand, solar drying, and synthetic insecticides such as phosphine [6,7]. However, many of these storage preservation methods are either ineffective, not scalable, or present risks. For instance, while effective, the use of chemical insecticides presents certain drawbacks, such as adverse health and environmental hazards, and insect resistance [8,9,10]. Consequently, there have been efforts to search for alternative approaches to manage insect pests of stored products.

Hermetic storage bags, including the Purdue Improved Crop Storage (PICS), are chemical-free storage technologies that effectively minimize losses caused by *C. maculatus* [6,11]. Insects on grain stored in hermetic containers naturally deplete the intergranular residual oxygen (O_2_) through respiration, leading to their death [12,13,14]. This process of oxygen depletion can take several days or even weeks to reach the 5% lethal level, depending upon the initial infestation level and environmental conditions such as temperature [15].

Grain damage or loss of quality may occur if the process of oxygen depletion is prolonged. Quality preservation becomes critical when dealing with products such as fortified maize or cassava (with provitamin A) that lose their nutritional value in the continued presence of oxygen, light, and heat [16]. These crops have been introduced in Africa as a strategy to supplement vitamin A for children [17,18]. These studies have identified storage as a critical area of intervention to maintain the quality of biofortified crops after harvest. Losses of carotenoids can occur after several months of storage and reach up to 60% or more [19,20]. Hermetic storage, used by smallholder farmers in Africa, has demonstrated limited effectiveness in mitigating these losses [19]. 

Several approaches are used to enhance the efficacy of hermetic storage. These include using oxygen scavengers or gases such as nitrogen and carbon dioxide to remove the residual oxygen [21,22,23]. Flushing nitrogen (N_2_) or CO_2_ into hermetic storage systems can speed up insect death compared to relying on natural processes such as insects and micro-organisms [24,25]. Oxygen scavengers can enhance the efficacy of hermetic storage, extend food shelf-life, and help maintain product quality [26,27]. A study conducted on biofortified maize found that, after 4 months of storage, carotenoid content was significantly higher in grain stored in PICS bags with oxygen scavengers than those without them or in common polypropylene woven bags [28]. Though effective, these approaches are nonexistent (unavailable) and unsuitable (impractical and costly) on smallholder farms in developing countries.

In the quest to enhance food preservation, exploring alternative solutions tailored to smallholder farms is imperative. Prior research has shed light on these solutions. For instance, placing soaked soybean seeds into stored grains reduced oxygen levels to 1% within 8 days [29]. Similarly, germinating cowpea seeds kept into empty jars effectively depleted oxygen to less than 1% level within just 24 h [29]. However, despite these insights, there remains a limited understanding of the broader implications of germinating seeds on oxygen depletion, insect pests, and grain quality. We hypothesized that variations in germinating seeds will lead to different levels of hypoxia and insect mortality. This study assessed the effects of (i) germinating seeds on oxygen depletion and grain quality and (ii) hypoxia induced by germinating seeds on adult insect mortality, egg counts, and progeny development.

## 2. Materials and Methods

### 2.1. Seed Preparation and Germination

Untreated seeds of black-eyed cowpea (*Vigna unguiculata*) were obtained from L.A. Hearne company (King City, CA, USA). Seeds in good condition with intact seed coats were selected. Medium-sized seeds, weighing an average of 320 ± 10 mg/seed, were used for the experiment. Approximately 1700 seeds were soaked to ensure enough germinating seeds were available on the day of the experiment.

Cowpea seeds were sterilized for 5 min in a solution containing 50% household bleach (7.5% sodium hypochlorite) with 50% distilled water to prevent fungal growth. Subsequently, all seeds were thoroughly washed with distilled water multiple times and allowed to air-dry at room temperature for half an hour. Following air drying, seeds without seed coats or split cotyledons were removed. 

After sterilization, 25 seeds were placed in 120 mL jars filled with 50 mL of distilled water and left for 24 h to imbibe. After imbibition, the seeds were left to germinate for four days on two layers of moistened Whatman no. 1 filter paper in sterile Petri dishes. The Petri dishes were kept in the dark at room temperature (25 ± 2 °C) until the desired germination stage. The filter paper was regularly moistened with distilled water to maintain moisture. Each day, any ungerminated or dead seeds were removed from the Petri dish to prevent fungal growth. Four days following imbibition, well-germinated seeds were selected for oxygen depletion. This germinating seed stage depletes oxygen faster, is quickest to achieve, and is easy to handle [29]. 

### 2.2. Insect Rearing

The insects used in this experiment were reared in a Conviron insect growth chamber (Model CMP4030; CONVIRON., Winnipeg, MB, Canada). The cowpea grains (L.A. Hearne Company, King City, CA, USA), stored at room temperature, were used to rear the insects. Colonies were set up approximately a month before the start of the experiment. Adults *C. maculatus* were removed from a pre-established colony using a vacuum aspirator and transferred to one-liter jars containing clean cowpea grains. Insects were allowed to breed and lay eggs for 48 h and removed. After removing the adults, the grain was held in a growth chamber at 25 ± 1 °C and 40 ± 5% relative humidity (RH) until new adults emerged. The newly emerged adults (within 3 days) were separated from the grains using a number 6 sieve, and then 10 insects (5 female and 5 male) were moved into a 30 mL round wide-mouth plastic jar. Female and male cowpea bruchids were identified by examining the shape of the abdomen and markings on the elytra. Females are identified by a larger, black-marked plate at the end of the abdomen, along with an elytra longer than 1 mm, giving them an ovoid shape. In contrast, males have a smaller, stripe-less plate and elytra shorter than 1 mm, resulting in a rounded appearance [30,31,32].

### 2.3. Experimental Setup and Design

Treatments consisted of 10, 20, and 30 germinating seeds. The volume and number of germinating seeds were determined based on preliminary experiments. The germinating seeds were placed in 30 mL vials (Wheaton Glass Sample bottle, CP Lab Safety, Novato, CA, USA) with perforated lids, each containing 10 seeds. Similarly, 10 insects were placed in 30 mL round wide-mouth plastic vials with perforated lids containing 30 cowpea grains for laying eggs. The vials with insects and those with seeds (based on the treatment) were placed in 2 L jars filled with cowpeas (Figure S1). On average, a 2 L jar filled with cowpeas had about 149 mL of intergranular residual oxygen available. Each treatment jar was hermetically sealed with two layers of Parafilm M^®^ Laboratory film inside and outside the lids, while control jars were not sealed hermetically. There were sets of jars (4 replicates for each treatment) opened at 24, 48, 72, 96, and 120 h. Four jars containing 0 germinating seeds and 10 insects each were included in the experiment to serve as the control. These jars were not hermetically sealed and were assessed at the 5 opening times. There were 60 jars (3 treatments, 4 replicates, and 5 opening times) filled with cowpea grains containing germinating cowpea seeds and insects, along with 4 jars (control) containing only insects and cowpea grains.

### 2.4. Data Collection

#### 2.4.1. Oxygen, Temperature, and Relative Humidity

Oxygen levels were monitored for 5 days using an OxySense^®^ 525OI Oxygen Analyzer device (Industrial Physics, Devens, MA, USA). The oxygen concentrations were measured at intervals of 3 h for the first 6 h, then at 6 h intervals until 30 h, and once a day thereafter. A repeated measurement study design was used to record changes in oxygen concentration within each jar over time. This provided an insight into how oxygen levels fluctuated over time. USB data loggers (Lascar, Erie, PA, USA) were kept only inside hermetic jars opened after 120 h to monitor RH and temperature. Three data loggers were placed outside the jars to monitor the ambient atmosphere of the room (laboratory). The data loggers were programmed to record RH and temperature data every hour.

#### 2.4.2. Adult Mortality

Treatments were opened at intervals of 24 h each day to collect data (destructive sampling except for the control). During each opening, cowpea grains and a jar containing insects were taken out of each container while the germinating seeds were discarded. The jar containing insects was emptied onto white paper to determine the survival of *C. maculatus* adults. If an adult *C. maculatus* moved upon exposure to normal oxygen, it was considered alive and transferred to a separate container. Inactive adults were gently touched with a camel-hair brush, and if they showed any movement in response, they were recorded as alive. Lastly, *C. maculatus* adults that did not respond to the touching were placed back in a growth chamber with a temperature of 25 ± 1 °C and a relative humidity of 40 ± 5% for an additional 2 h. After this period, insects were reassessed to ensure they were dead and not in hypoxic stress.

#### 2.4.3. Egg Count

After evaluating the mortality of adult insects, both the cowpea grains and the adults were separated. The eggs were then counted by inspecting the surface of each grain under appropriate lighting and using a hand magnifier. Eggs laid by a single female were calculated by dividing the total number of eggs recorded in a single jar by the total number of females (5 females) present in the jar. 

#### 2.4.4. Progeny Development

After counting, the cowpea grains with eggs for each treatment were incubated in a Caron insect growth chamber (Model 6025-1, 115 VAC, Caron Growth chambers, Marietta, OH, USA) at 25 ± 1 °C and 40 ± 5% RH. After 35 days of incubation, insect adults that emerged were recorded every day for up to two weeks for each treatment and control. 

#### 2.4.5. Moisture Content 

The moisture content of stored cowpeas (not germinating seeds) was assessed at the beginning and the end (jars opened on the fifth day) of the experiment using the oven-dry method at 103 °C for 72 h. Three 15 g samples were used to measure moisture content.

### 2.5. Statistical Analysis 

Since data for the time variable depict a non-normal pattern, a log transformation of the time variable was performed before the regression analysis. A linear regression model was fitted to examine the relationship between oxygen levels and the variables log(time) (logarithm of respiration rate) and treatment. 

The estimated multiple linear regression equation is expressed as follows:

Oxygen concentration (%) = β_0_ + β_1_ log(time) + β_2_ Treatments + β_3_ log(time) * Treatments + ε

whereas

β_0_ = intercept
β_1_ = slope of log(time)
β_2_ = slope of Treatments
β_3_ = slope of interaction term of log(time) and Treatments
ε = Error term

The multiple regression model with interaction terms for single combinations is as follows:

Oxygen concentration (%) = β_0_ + β_1_ log(time) + β_2_ 1(Treatments = 10 seeds) + β_3_ 1(Treatments = 20 seeds) + β_4_ 1(Treatments = 30 seeds) + β_5_ 1(Treatments = 10 seeds) log(time) + β_6_ 1(Treatments = 20 seeds) log(time) + β_7_ 1(Treatments = 30 seeds) log(time) + ε

1 (Treatments = 10 seeds) means it’s 1 when Treatment = 10 seeds else it’s 0.

10 seeds y = (β_0_ + β_2_) + (β_1_ + β_5_) log(time) + ε (1)

20 seeds y = (β_0_ + β_3_) + (β_1_ + β_6_) log(time) + ε (2)

30 seeds y = (β_0_ + β_4_) + (β_1_ + β_7_) log(time) + ε (3)

The evaluation of the models involved the calculation of estimated marginal means (EMMs) using the “emmeans” package [33]. The “emtrends” function from the same package was utilized to analyze slopes. Post hoc pairwise comparisons were conducted to examine the differences between treatments further. 

Analysis of variance was conducted to compare the means of oxygen concentration, mean percentage mortality, average egg counts, adult emergence, and mean moisture content. Means were separated using Tukey adjustments at a 95% confidence level. The correlation coefficient was estimated among six variables: number of germinating seeds, oxygen, egg count, opening time, mortality, and progeny development. All statistical analyses were performed using R 4.3.2 and Microsoft Excel 2016.

## 3. Results

### 3.1. Oxygen Concentration

**Figure 1:**
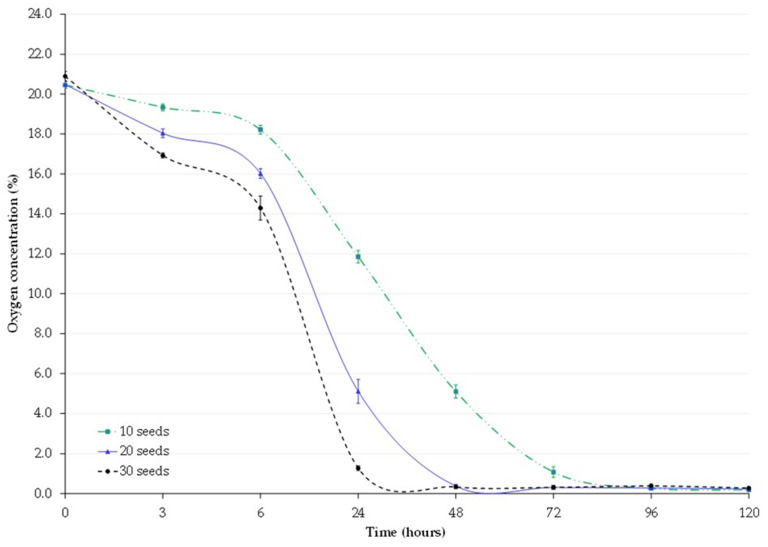
Oxygen depletion by 10, 20, and 30 germinating cowpea seeds (over 120 h) inside 2 L hermetic jars filled with cowpea and containing insects. Each error bar represents a standard error of the mean (SEM, *n* = 4).

All three treatments exhibited different behaviors of oxygen depletion. The treatment of 30 germinating seeds consistently showed significantly higher oxygen depletion rates compared to 10 and 20 germinating seeds (Figure 1). The mean oxygen level fell to about 5% within 48 h in all treatments. It took less than 24 h for 30 seeds to reach an oxygen level below 5%, 24 h for 20 seeds, and 48 h for 10 seeds to reach the threshold (Table 1). Oxygen depletion was affected by treatments (F = 1759.98, *p* < 0.001) and time (F = 322.18, *p* < 0.001). No changes in oxygen levels were observed in the non-hermetic jars (graph not shown). The multiple linear regression model results support the findings in Figure 1 and Table 2. The slope estimates from 0 to 24 h, using data from all treatment jars of the respective treatments (total 20 jars), indicated a significant interaction between exposure time and treatments (F = 86.81, *p* < 0.001) with an R^2^ of 70.56%. The highest coefficient was found in the treatment of 30 seeds (Table 2). Pairwise comparison at a 95% confidence interval revealed that the slope of all three treatments significantly differed (*p* < 0.05). A marginal slope estimate was generated by plugging the data from Table 2 into the previously described equation for each treatment (Equations (1) to (3)). This estimate reveals that the hermetic storage jars with 10, 20, and 30 germinating seeds were expected to experience a reduction in oxygen level by −0.55, −1.03, and −1.39 units for every additional hour of exposure, respectively.

### 3.2. Adult Mortality

Insect mortality was affected by treatments (F = 288.04, *p* < 0.001) and exposure time (F = 168.23, *p* < 0.001). Complete mortality was noted in all three treatments but occurred at different times (Table 3). A 100% adult mortality result was achieved in the treatments with 20 and 30 germinating seeds within 72 h. However, in the treatment with 10 seeds, complete mortality was observed only after 120 h. While complete death was observed on the same day of opening for both 20 and 30 germinating seeds, a distinction in the mortality rate emerged. After 48 h, the treatment with 30 germinating seeds exhibited an 85% mortality, in contrast to the 25% observed in the treatment with 20 germinating seeds.

### 3.3. Egg Count

**Figure 2:**
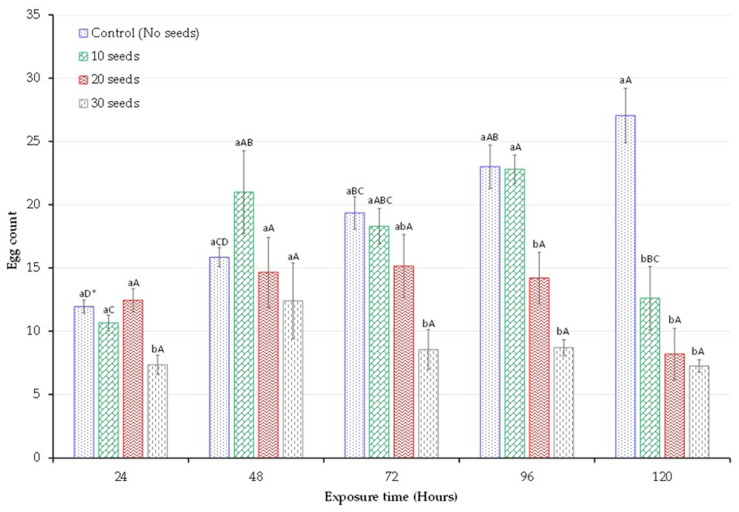
Average number of eggs laid by a single female cowpea weevil after 24, 48, 72, 96, and 120 h. Treatments consisted of 10, 20, and 30 germinating seeds in 2 L jars and control with no germinating cowpea seeds. * Means within the same hour (lower case) and the same treatment (upper case) followed by the same letter are not significantly different (*p* < 0.05). Error bars represent a standard error of the mean (SEM, *n* = 4).

Figure 2 provides an insight into the influence of hypoxia from germinating seeds on the total number of eggs laid by cowpea bruchid females. The number of eggs laid by a female cowpea bruchid was impacted by treatments (F = 33.61, *p* < 0.001) and exposure time (F = 9.77, *p* < 0.0009). Within the treatments, the egg counts over the 5 days remained consistently the same for both the 20 and 30 germinating seeds (Figure 2). However, the 10 germinating seeds exhibited higher egg counts at 96 h compared to both the 20 and 30 germinating seeds. In contrast, the control had gradually increased egg counts over time. 

When comparing the treatments with the control on each day of observation, distinct patterns were noticed. At 24 h, the treatment with 30 germinating seeds exhibited a lower egg count in comparison to the other treatments and control. However, after 120 h of opening, all three treatments exhibited lower egg counts when compared to the control (Figure 2). Prolonged exposure of female cowpea weevils to hypoxia induced by germinating seeds limited their ability to lay more eggs. This is demonstrated in a negative correlation between egg laying and adult exposure time (r = −0.58).

### 3.4. Progeny Development

Adult emergence was suppressed by treatments (F = 1895.79, *p* < 0.001) and exposure time (F = 623.43, *p* < 0.001). The control had higher adult emergence than the treatments for all exposure times (Table 4). Across all treatments, the percentage of adult emergence decreased with longer exposure time. In the treatments with 20 and 30 germinating seeds, no adults emerged from eggs laid after 120 and 96 h of exposure to hypoxia, respectively. However, in the treatment with 10 germinating seeds, there was still 0.29% adult emergence from eggs laid after 120 h of exposure to hypoxia.

### 3.5. Moisture Content

The initial moisture content of cowpea grain was 8.9% but increased to above 9% in the treatments and the control (Table 5). The moisture content of the stored cowpea grains was affected by exposure time (F = 14.78, *p* < 0.031) but not treatments (F = 0.99, *p* < 0.439). There was no interaction between treatments and exposure time (F = 0.90, *p* < 0.477). No significant differences in moisture content were observed in the treatments and control at the end of the experiment. However, the moisture contents were significantly higher between the initial and the fifth day for only 30 germinating seeds and the control.

### 3.6. Temperature and Relative Humidity

**Figure 3:**
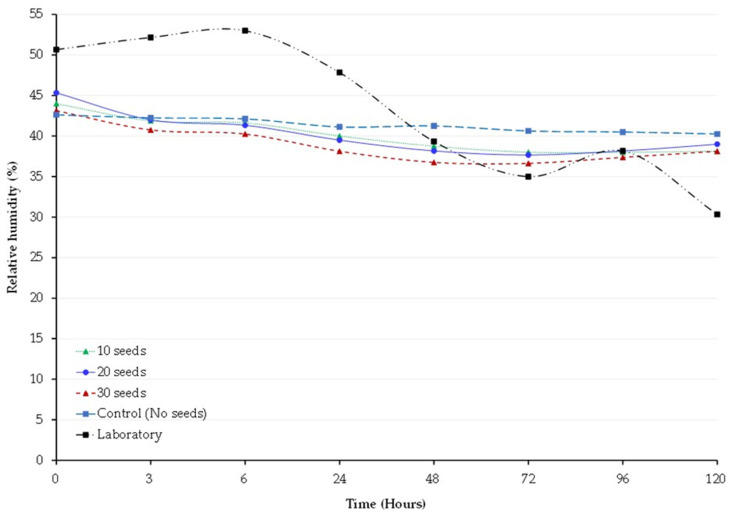
Temporal fluctuations in relative humidity inside hermetic jars filled with cowpea grain and containing 10, 20, or 30 germinating seeds, control, and laboratory for 120 h.

The average RH inside the 2 L hermetic jars storing cowpeas was 40 ± 0.79, 40.14 ± 0.92%, and 38.8 ± 0.81% for 10, 20, and 30 germinating seeds, respectively, and 41.5 ± 0.32% for the non-hermetic jars or control. Relative humidity was relatively constant during the 5 days in treatments and control (Figure 3). No differences were observed. However, the RH of the room fluctuated (43.3 ± 3.07%) during the experiment. The temperature was 24 ± 1.5 °C throughout the experiment regardless of germinating seed treatments, control, and the room.

## 4. Discussion

In this study, we assessed how germinating cowpea seeds affect oxygen depletion in hermetically sealed jars filled with grains and containing insects. All three different treatments showed varying rates of oxygen depletion. A higher slope was observed in treatments with a higher number of seeds. Oxygen consumption increased by 1.87 and 2.53 with the augmentation of germinating seeds from 10 to 20 and 30, respectively. This finding is not surprising because as the number of germinating seeds increased, the oxygen depletion rate also increased. While depletion rates varied among treatments, all of them eventually reached oxygen levels of 1% or below within 72 h. Swiftly achieving anoxic conditions is crucial to prevent further insect development and the deterioration of food quality. These results demonstrate that the presence of a few germinating seeds can efficiently deplete oxygen in 2 L jars to lethal levels within a mere 3 days of exposure. Germinating seeds were as effective or even better than other mechanisms such as insects [34], gases [21], and soaked seeds [35] mediated oxygen removal in modified or controlled atmospheres. 

Hypoxia induced by germinating seeds significantly affected adults of *C. maculatus,* resulting in complete mortality between 3–5 days of exposure. A higher number of germinating seeds led to quicker oxygen depletion and earlier adult mortality. Similar adult mortality (within 4 days) was observed when oxygen levels were maintained at 1% using nitrogen [22,36]. Faster oxygen depletion meant that insects experienced prolonged exposure to lower hypoxia, accelerating adult mortality. There was a strong positive Pearson correlation of 0.81 (*p* > 0.01) between exposure time and insect mortality, indicating higher mortality rates as exposure time increased. Germinating seeds were as effective as other controlled atmosphere methods (i.e., nitrogen) in accelerating insect deaths. Lower oxygen levels, below 3% and preferably less than 1%, generally lead to higher insect mortality rates, especially when rapid pest elimination is required [37,38,39,40].

Furthermore, the hypoxic conditions created by germinating seeds impacted the behavior of female cowpea weevils in laying eggs. It is well documented that insects cease feeding and reproducing when oxygen levels reach a lethal threshold [14]. This explains why, in the treatments involving 20 and 30 seeds, egg counts remained constant throughout the entire observation period. In both cases, the oxygen levels dropped to 5% or lower within 24 h, effectively ceasing further egg laying by female *C. maculatus*. These findings are corroborated by prior research, which demonstrated reduced egg laying by female cowpea weevils when exposed to hypoxia [25,41]. Similar behavior of reduced female egg laying and a slowdown in embryonic development were observed when insects were exposed to 13% CO_2_ levels [42]. A negative Pearson correlation of −0.58 (*p* > 0.01) was observed between eggs laid and adult exposure time. Extended exposure of female cowpea weevils to hypoxia induced by germinating seeds diminished their ability to lay eggs. 

Low oxygen levels not only affected adult mortality and egg laying but significantly suppressed the development of progeny. No adults emerged within 50 days post-treatment after insects were exposed to hypoxia from 20 and 30 germinating seeds for 120 and 96 h, respectively. Previous research showed that exposure of insects to low oxygen (e.g., 1%) for a few days resulted in no adult emergence within 45 days post-treatment [22,25]. Similar results were achieved using other gases, such as CO_2_. Studies reported complete suppressions of progeny development of *Sitophilus* spp. in 30 days and *Sitotroga cerealella* (Olivier) in 11 days under CO_2_ atmospheres [43,44]. A negative Pearson correlation of −0.64 (*p* > 0.01) was observed between progeny development and exposure time, indicating that as exposure time increased, offspring development notably declined. Research findings have demonstrated that the emergence of adult insects following exposure to hypoxia is influenced by factors such as insect life stage, oxygen level, and exposure time [24,45]. 

Moisture content significantly increased only for 30 germinating seeds and control when comparing the initial and final measurements. However, no differences between the three treatments and the control group were observed at the end of the experiment. The RH had a marginal effect on moisture content with an increase of 0.23, 0.20, and 0.41 percentage points for 10, 20, and 30 germinating seeds, respectively. The significant difference between the initial and final moisture contents for 30 germinating seeds may be explained by RH released by the higher number of seeds compared to 10 and 20 germinating seeds. A higher humidity level caused by 30 germinating seeds could have been absorbed by the stored grains, potentially masking its effects. In control, the increase in moisture content might be explained by rapid absorption of the humidity from the room, which was higher within the first 24 h. An increase in grain moisture under higher RH is well documented and could be conducive to mold growth and subsequent grain deterioration [46,47]. Further research is needed to explore ways of minimizing the release of RH by germinating seeds into stored grains.

## 5. Conclusions

The findings of this study suggest that germinating seeds can be effectively used as oxygen scavengers and significantly affect insect mortality, egg laying, and progeny development in hermetic storage systems. Despite the differences in depletion rates, all treatments achieved lethal oxygen levels of 1% or below within 72 h of exposure. The higher number of germinating seeds reached lower oxygen levels more rapidly, resulting in early adult mortality. The overall complete mortality was achieved between 3 and 5 days of exposure. The hypoxic conditions created by germinating seeds also suppressed egg laying and insect progeny development. Grain moisture content slightly increased in all treatments, but the quality was not compromised. Germinating seeds are as effective as other controlled atmospheres (e.g., gases such as nitrogen) in accelerating insect deaths. However, further research is needed to assess (i) the use of germinating seeds in larger hermetic storage containers (e.g., the number of seeds needed, and time required to keep them inside hermetic containers) and (ii) ways of minimizing the effect of germinating seeds on grain quality (e.g., moisture content and germination). 

## Figures and Tables

**Table 1:** Average oxygen concentration (%) in hermetic and non-hermetic (control) jars after 24, 48, 72, 96, and 120 h when germinating cowpea seeds were used as oxygen scavengers. Treatments consisted of 10, 20, and 30 germinating cowpea seeds in 2 L jars, and control had no germinating seeds.

	Residual Oxygen Level (%, Mean ± SEM)					
	Time					
Treatment	6 h	24 h	48 h	72 h	96 h	120 h
10 Seeds	18.22 ± 0.21 b *	11.85 ± 0.32 b *	5.10 ± 0.33 b	1.08 ± 0.27 b	0.27 ± 0.02 b	0.19 ± 0.02 b
20 Seeds	16.02 ± 0.24 c	5.11 ± 0.60 c	0.38 ± 0.09 c	0.31 ± 0.03 b	0.29 ± 0.06 b	0.24 ± 0.04 b
30 Seeds	14.39 ± 0.60 d	1.28 ± 0.12 d	0.34 ± 0.09 c	0.32 ± 0.09 b	0.39 ± 0.09 b	0.28 ± 0.06 b
Control	21.36 ± 0.90 a	21.20 ± 0.93 a	20.89 ± 0.96 a	20.6 ± 0.86 a	20.92 ± 0.94 a	20.87 ± 0.85 a

* All data are mean ± standard error of mean (SEM). Under the same time, means within the same column followed by the same letter are not significantly different (*p* < 0.05).

**Table 2:** Intercepts, slopes for regression equations, and interaction with log(time) for 10, 20 and 30 germinating cowpea seeds.

Variables *	Estimate	Std. Error	t Value	Pr(>|t|)
(Intercept) (β_0_)	21.37	0.42	50.30	<0.001
log_hr (β_1_)	−0.008	0.11	−0.08	0.94
10 seeds (β_2_)	−3.62	0.60	−6.02	<0.001
20 seeds (β_3_)	−6.43	0.60	−10.70	<0.001
30 seeds (β_4_)	−8.42	0.60	−14.01	<0.001
log_hr: 20 seeds (β_5_)	−0.55	0.15	−3.63	<0.001
log_hr: 20 seeds (β_6_)	−1.03	0.15	−6.75	<0.001
log_hr: 30 seeds (β_7_)	−1.39	0.15	−9.11	<0.001

* Variables consisted of germinating seeds: β_0_ = intercept, β_1_ = slope of log(time), β_2_ = slope of 10 germinating seeds, β_3_ = slope of 20 germinating seeds, β_4_ = slope of 30 germinating seeds, β_5_ = slope of interaction between log(time) and 10 germinating seeds, β_6_ = slope of interaction between log(time) and 20 germinating seeds, β_7_ = slope of interaction between log(time) and 30 germinating seeds. Treatments consisted of 10, 20, and 30 germinating cowpea seeds in 2 L jars.

**Table 3:** Average mortality (%) of *C. maculatus* adults in cowpea stored in hermetic and non-hermetic (control) containers after 24, 48, 72, 96, and 120 h when germinating cowpea seeds were used as oxygen scavengers. Treatments consisted of 10, 20, and 30 germinating cowpea seeds in 2 L jars, and control had no germinating seeds.

Adult Mortality (%, Mean ± SEM)					
Exposure Time					
Treatment	24 h	48 h	72 h	96 h	120 h
10 Seeds	5 ± 2.89 abC *	7.5 ± 2.5 cBC	30 ± 10.8 bB	97.5 ± 2.5 aA	100 ± 0 aA
20 Seeds	10 ± 7.07 abC	25 ± 6.45 bB	100 ± 0 aA	100 ± 0 aA	100 ± 0 aA
30 Seeds	12.5 ± 4.79 aC	85 ± 6.45 aB	100 ± 0 aA	100 ± 0 aA	100 ± 0 aA
Control	0 ± 0 bA	5 ± 5 cA	7.5 ± 4.79 cA	7.5 ± 4.79 bA	10 ± 4.08 bA

* All data are mean ± standard error of mean (SEM). Means within the same column (lower-case letter) and the same row (upper-case letter) followed by the same letter are not significantly different (*p* < 0.05).

**Table 4:** Average adult emergence (%) of *C. maculatus* 50 days post-treatment after exposure to normoxia. Cowpea were stored in hermetic and non-hermetic (control) containers for 24, 48, 72, 96, and 120 h when germinating cowpea seeds were used as oxygen scavengers. Treatments consisted of 10, 20, and 30 germinating cowpea seeds in 2 L jars, and control had no germinating seeds.

	Adult Emergence (%, Mean ± SEM)				
	Exposure Time				
Treatment	24 h	48 h	72 h	96 h	120 h
10 Seeds	64.92 ± 2.99 bB *	79 ± 1.38 aA	30.89 ± 1.19 bC	14.78 ± 1.57 bD	0.29 ± 0.29 bE
20 Seeds	63.85 ± 1.54 bA	40.66 ± 2.89 bB	8.88 ± 1.72 cC	2.54 ± 1.48 cD	0 ± 0 bE
30 Seeds	50.09 ± 1.78 cA	6.53 ± 1.40 cB	0.40 ± 0.40 dC	0 ± 0 cC	0 ± 0 bC
Control	83.24 ± 0.5 aABC	86.13 ± 1.31 aAB	82.92 ± 2.82 aBC	76.03 ± 1.93 aC	90.94 ± 1.51 aA

* All data are mean ± standard error of mean (SEM). Means within the same column (lower case letter) and the same row (upper case letter) followed by the same letter are not significantly different (*p* < 0.05).

**Table 5:** Average moisture content (%) of cowpea stored in hermetic and non-hermetic (control) containers at the beginning and end of the experiment when germinating cowpea seeds were used as oxygen scavengers. Treatments consisted of 10, 20, and 30 germinating cowpea seeds in 2 L jars, and control had no germinating seeds.

Treatment	Initial	5 Days
10 seeds	8.90 ± 0.1 aA *	9.13 ± 0.09 aA
20 seeds	8.90 ± 0.1 aA	9.10 ± 0.06 aA
30 seeds	8.90 ± 0.1 aB	9.31 ± 0.03 aA
Control	8.90 ± 0.1 aB	9.25 ± 0.10 aA

* All data are mean ± standard error of mean (SEM). Means within the same column (lower case letter) and the same row (upper case letter) followed by the same letter are not significantly different (*p* < 0.05).

## Data Availability

Raw data are not publicly available but may be obtained upon request.

## Supplementary Materials

The following supporting information can be downloaded at: https://www.mdpi.com/article/10.3390/insects14120954/s1, Figure S1: Cowpea stored in 2 L hermetic jar containing 30 mL vials (with germinating seeds or insects) and a data logger.
