# Supplementary material for: Enhancing Airtight Storage with Germinating Cowpea Seeds: Impacts on Insect Mortality, Progeny and Grain Quality
Source: Insects. 2023 Dec 15;14(12):954. doi: 10.3390/insects14120954 (PMC10744301; doi:10.3390/insects14120954)
Supplement: Supplementary file 1 [file insects-14-00954-s001.zip › insects-2611912-Figure S1.pdf]

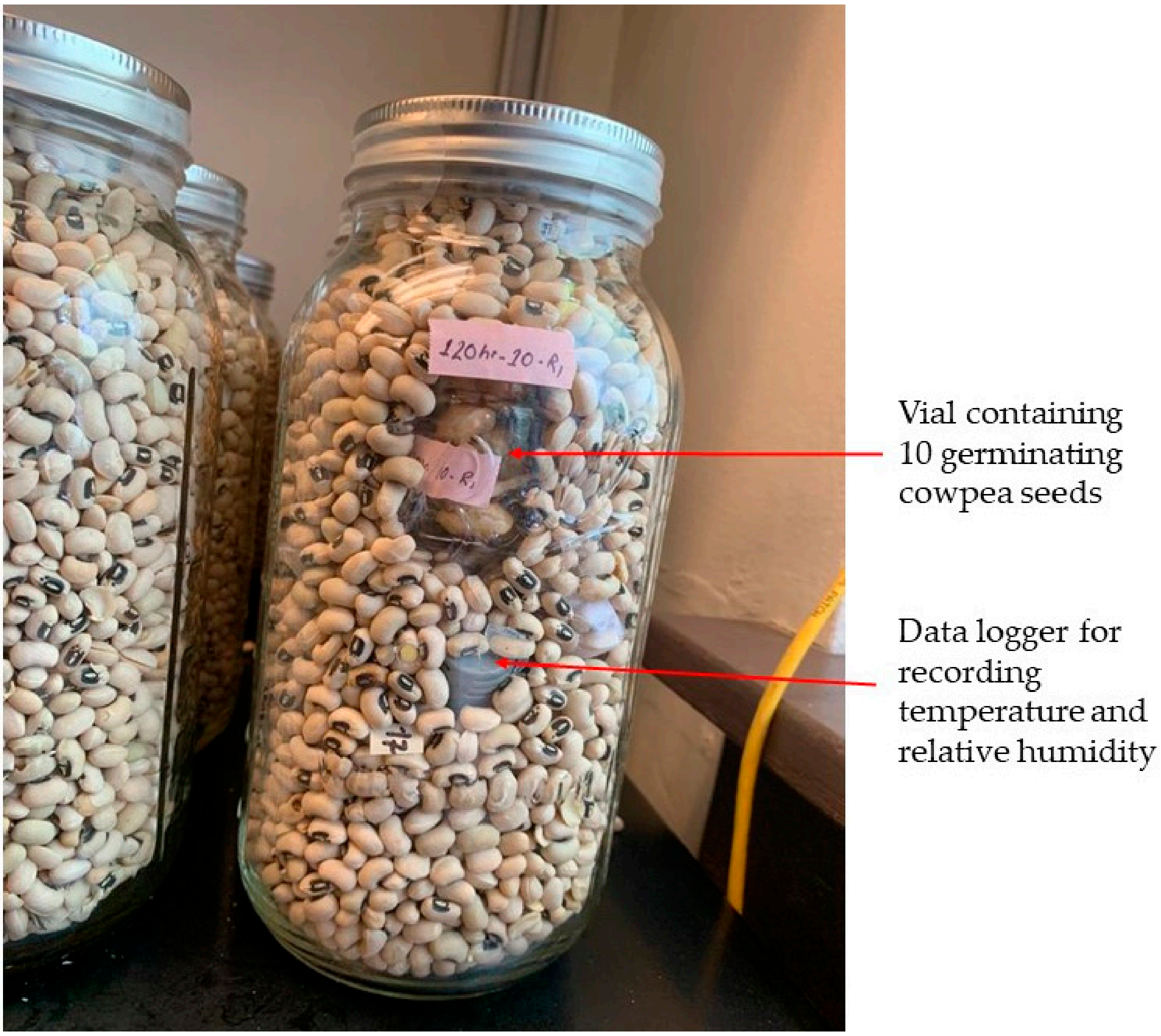

Figure S1. Cowpea stored in 2 L hermetic jar containing 30 mL vials (with germinating seeds or insects) and a data logger.
